# Integrating observational and modelled data to advance the understanding of heat stress effects on pregnant subsistence farmers in the gambia

**DOI:** 10.1038/s41598-024-74614-y

**Published:** 2024-10-23

**Authors:** Carole Bouverat, Jainaba Badjie, Tida Samateh, Tida Saidy, Kris A. Murray, Andrew M. Prentice, Neil Maxwell, Andy Haines, Ana Maria Vicedo Cabrera, Ana Bonell

**Affiliations:** 1 Oeschger Center for Climate Change Research, University of Bern, Bern, Switzerland; 2 Institute of Social and Preventive Medicine, University of Bern, Bern, Switzerland; 3 Medical Research Unit The Gambia, London School of Hygiene and Tropical Medicine, London, England; 4 Environmental Extremes Laboratory, University of Brighton, Brighton, England; 5 Centre On Climate Change and Planetary Health, London School of Hygiene and Tropical Medicine, London, England

**Keywords:** Maternal health, Climate change, Heat stress, Heat strain, Humidity, Climate change adaptation, Environmental health, Environmental health, Risk factors

## Abstract

Studies on the effect of heat stress on pregnant women are scarce, particularly in highly vulnerable populations. To support the risk assessment of pregnant subsistence farmers in the West Kiang district, The Gambia we conducted a study on the pathophysiological effects of extreme heat stress and assessed the applicability of heat stress indices. From ERA5 climate reanalysis we added location-specific modelled solar radiation to datasets of a previous observational cohort study involving on-site measurements of 92 women working in the heat. Associations between physiological and environmental variables were assessed through Pearson correlation coefficient analysis, mixed effect linear models with random intercepts per participant and confirmatory composite analysis. We found Pearson correlations between r-values of 0 and 0.54, as well as independent effects of environmental variables on skin- and tympanic temperature, but not on heart rate, within a confidence interval of 98%. Pregnant women experienced stronger pathophysiological effects from heat stress in their third rather than in their second trimester. Environmental heat stress significantly altered maternal heat strain, particularly under humid conditions above a 50% relative humidity threshold, demonstrating interactive effects. Based on our results, we recommend including heat stress indices (e.g. UTCI or WBGT) in local heat-health warning systems.

## Introduction

Pregnant women who work outdoors are particularly vulnerable to heat stress and face an elevated risk of heat-related adverse health outcomes^1–3^. Heat stress indices allow modelling of the heat transfer to the human body as effective temperature scales through the interplay of environmental variables such as air temperature, humidity, solar radiation, and air velocity^4^. More than 100 heat stress indices have been developed^5^. Assessing their appropriateness to a given study setting is key to produce robust and reliable predictions for stakeholders to interpret, communicate, and potentially prevent the health impacts of climate change^6,7^. Heat strain is defined as the physiological response to heat stress and becomes apparent by changes in parameters such as heart rate and core temperature, which are known to be related to exposure to above-optimal temperatures. The extent to which environmental factors act singularly or in combination to affect maternal physiology, is unclear as this topic has been under-researched to date^8,9^. Therefore, a more thorough understanding of the effects of heat stress on maternal physiology in highly vulnerable populations could support the adoption of specific heat stress indices that would allow accurate public health messaging and so reduce the health risks of working in the heat.

In West Africa, rapid and widespread changes in climate exacerbate the frequency, duration, and severity of extreme heat^10^. The annual number of days above postulated dangerous heat thresholds is projected to increase from under 50 days between 1995 and 2005 to 50–150 days under a global warming scenario of 1.6 °C, respectively to 250–350 days under a global warming scenario of 4.4 °C by the end of the century^11^. Regional climate models project an additional increase in mean annual surface temperature between 0.9 to 4.8 °C in The Gambia by 2100, depending on the shared socio-economic pathway^12^. The resulting health inequalities are projected to become even more pronounced in the future^13^. Additionally, adverse pregnancy outcomes are disproportionally frequent in low-income countries^14^. 42% of stillbirths and 66% of maternal deaths worldwide occur in sub-Saharan Africa^15^. These statistics are related to the healthcare structure, limited access to resources, and also environmental factors including heat stress^16^.

The mechanisms by which heat stress affects human physiology are well documented in the literature^5^. Thermoregulatory responses are activated to reduce excess heat storage and cool the body through convection, radiation, or evaporation^17,18^. When thermoregulation is unable to compensate for heat stress, negative health effects can arise, ranging from dizziness, dehydration, thermal fatigue, heat syncope, muscle cramps, and rashes to organ damage and heat stroke^16,19^. A growing body of scientific evidence suggests that pregnancy increases vulnerability to the effects of extreme heat because fetal development is sensitive to alterations in the internal environment and because of the added heat burden of fetal growth from increased metabolism^8,16^. Various studies indicate that exposure to heat during pregnancy affects placental and endocrine functions and increases the probability of adverse pregnancy outcomes including pre-eclampsia, premature birth, stillbirth, and prolonged labour^2,3^. Nonetheless, the effect of environmental factors on the physiological parameters of pregnant women and the applicability of heat stress indices in pregnancy are unclear.

**Fig. 1:**
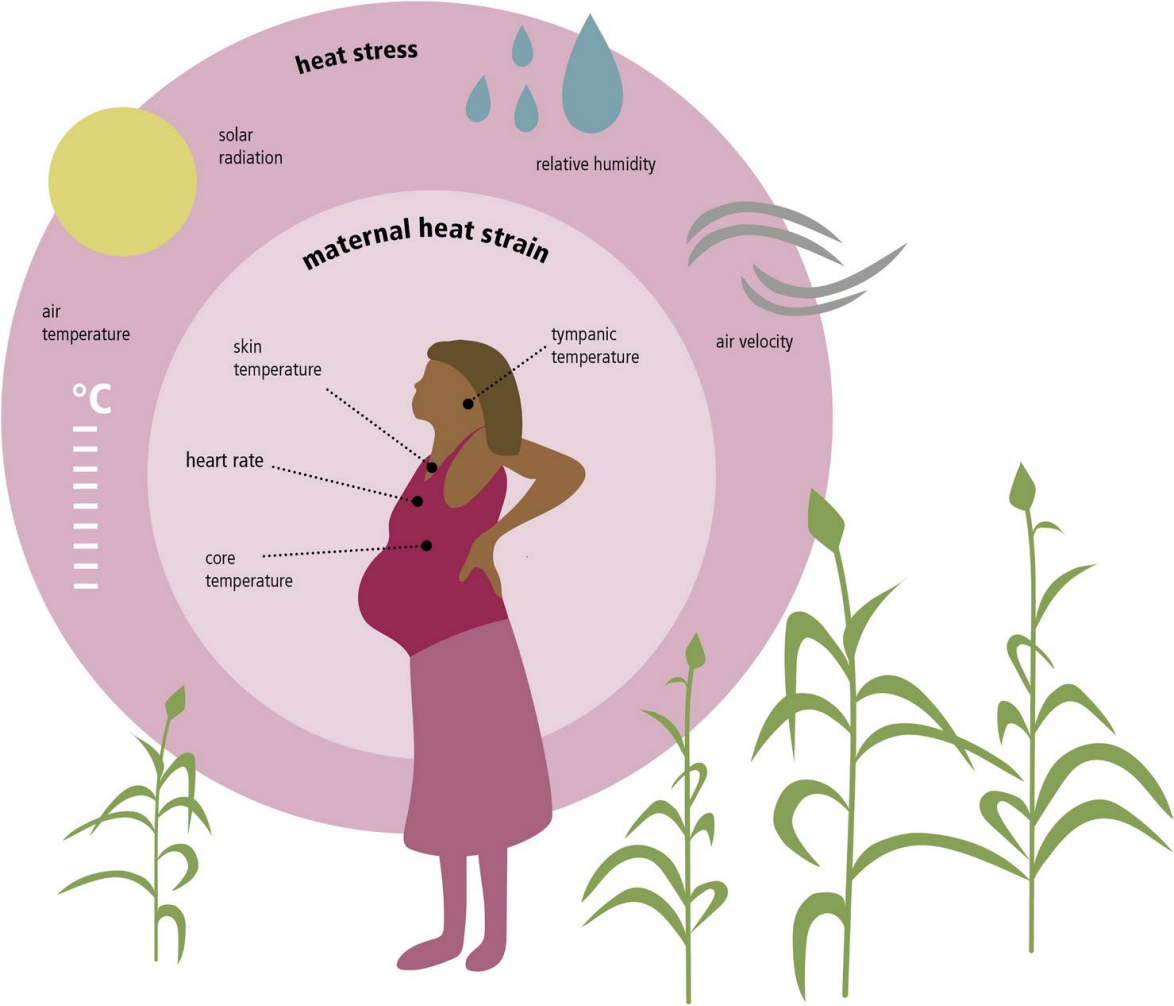
Visual overview of variables used in data analysis (own representation).

To address these gaps, we used data previously collected by Bonell et al.^1^ from 92 pregnant farmers in West Kiang, The Gambia in West Africa. The first aim was to identify the independent effects of environmental parameters on physiological parameters of agricultural workers during pregnancy. We included the following environmental parameters: air temperature, air velocity, relative humidity, black globe temperature, and modelled solar radiation (Fig. 1). The following physiological parameters were included: heart rate, skin temperature, tympanic temperature and estimated core temperature (Fig. 1). The second aim was to test the applicability of heat indices in our study context to determine the potential added value of incorporating these indices into local weather warning systems. We selected the Heat Index, Apparent Temperature, Wet-bulb Globe Temperature (WBGT), and Universal Thermal Climate Index (UTCI) based on the frequency of their use in the epidemiological literature on occupational health^5^,^20^ and maternal health^1,21^. The third aim was to investigate the compound effects of exposure to heat stress on heat strain as indicators for simultaneous changes in environmental and physiological variables. This study incorporated two types of data: (i) observational data, which contain both physiological and environmental on-site measurements of 92 pregnant women working in the heat in West Kiang, The Gambia, collected by the observational cohort study of Bonell et al.^1^ and (ii) modelled solar radiation data which was extracted from the ERA5 climate reanalysis^22^.

## Results

### Associations between environmental and physiological variables

**Fig. 2:**
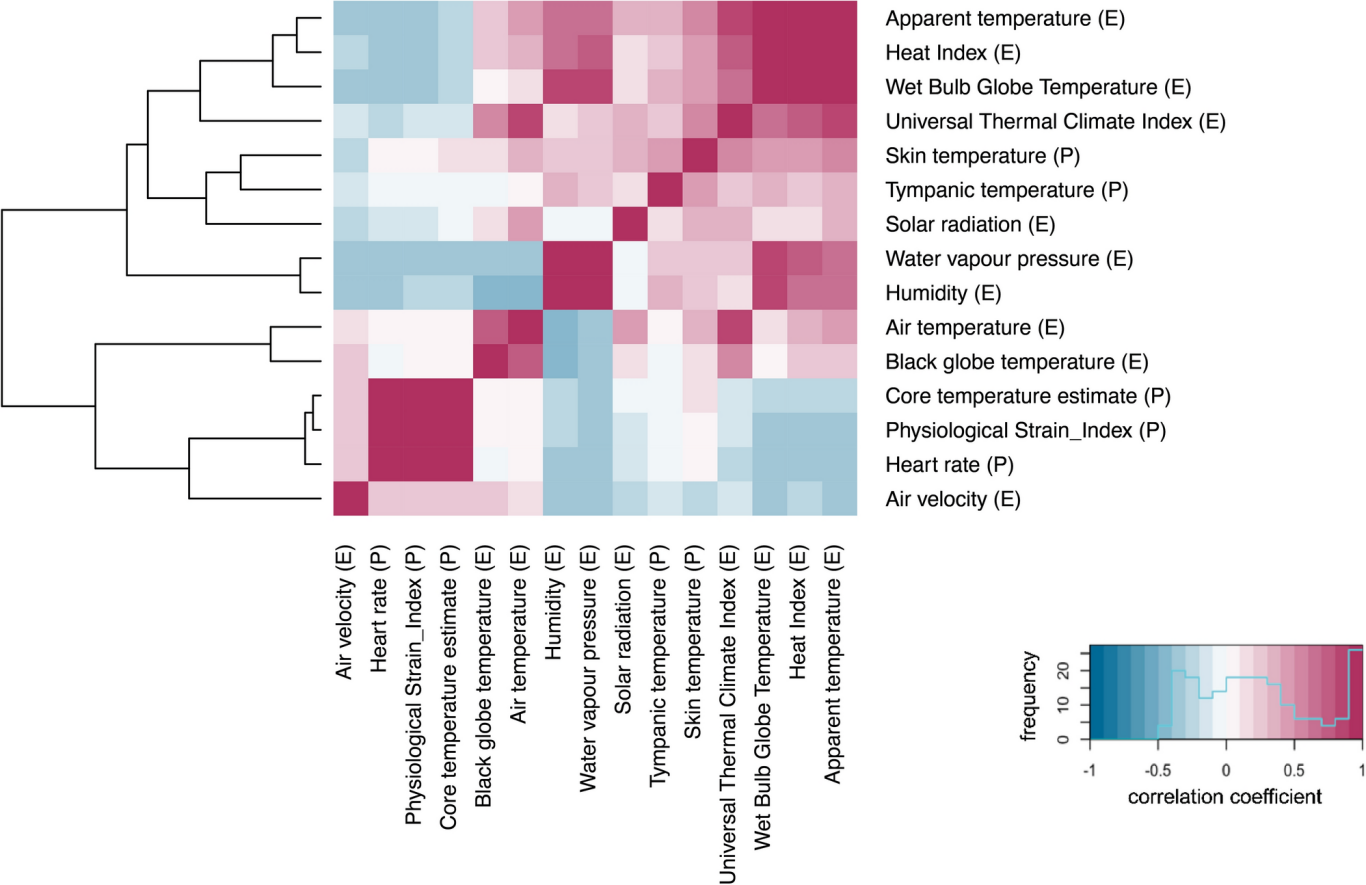
Heatmap indicating the strength of the Pearson correlation coefficients between variables as colour gradients. Denoted with (E) for environmental variables and (P) for physiological variables. *P*-values are contained in Supplementary Table 3. The dendrogram at the left side orders variables according to the similarity of their correlation with other variables.

Overall, Pearson product moment correlation coefficient analysis revealed interlinkages both within and between the pools of physiological and environmental variables. Visualized through the dendrogram lines at the left side of the heatmap (Fig. 2), we observed that, in terms of similarity in correlation pattern, the variables were not clustered in the initial two pools of environmental and physiological variables (Table 1). Instead, skin temperature and tympanic temperature were nested within the cluster of environmental variables, indicating that their associations followed patterns that were more similar to those of environmental variables than to those of other physiological variables.

**Table 1:** Descriptive statistics table of merged datasets with two main pools of variables.

Variable pool	Variable type	Variable	Unit	N	Mean	Std. Dev	Min	Pctl. 25	Pctl. 75	Max
Environmental variables	Observed	Air velocity	m/s	407	1.3	0.83	0.1	0.7	1.7	5.2
		Air temperature	°C	407	33	3.8	22	31	36	45
		Relative humidity	%	407	28	23	0	9.9	41	88
		Black globe temperature	°C	407	38	4.9	15	34	41	52
	Modelled	Solar radiation	J/m^2^	407	6e^6^	4.37e^6^	3.29e^5^	2.27e^6^	8.33e^6^	2e^7^
		Water vapour pressure	hPa	407	14	10	0	5	20	53
	Calculated	Universal thermal climate index	°C	407	33	3.9	20	31	35	51
		Apparent temperature	°C	407	33	4.4	19	31	36	51
		Heat index	°C	407	33	4.5	21	30	36	71
		Wet bulb globe temperature	°C	407	24	3.4	15	22	27	35
Physiological variables	Observed									
		Heart rate	bpm	353	106	13	65	97	115	147
		Skin temperature	°C	353	37	1.1	32	36	38	40
		Tympanic temperature	°C	89	37	0.35	36	37	37	38
	Calculated	Core temperature	°C	299	38	0.31	37	37	38	39
		Physiological strain index	NA	299	3.8	1	1.4	3.1	4.5	7.1
	Observed	Gestational age	weeks	407	27	6.9	12	23	33	41
		Fitness status, 6 min walking test	m	407	498	74	135	461	544	687
	Estimated	Metabolic rate	kcal/kg/h	357	3.2	0.85	1.9	2.3	3.8	5.2

Within the pool of environmental variables, Pearson correlation coefficients ranged from negligible to very high strength (− 0.08 < *r *< 0.80) (Fig. 2, Supplementary Table 2)^23^. Specifically, the heat stress indices, namely the UTCI, WBGT, the Heat Index, and Apparent Temperature, correlated with moderate to very strong strength (0.65 < *r *< 0.94), indicating their convergent validity (Supplementary Table 2). Convergent validity measures how closely an index is related to another index that measures the same concept^24^.

Within the pool of physiological variables, Pearson correlation coefficients ranged from negligible to very high strength (− 0.01 < *r* < 0.99) (Fig. 2, Supplementary Table 2). The tympanic temperature of pregnant women was positively associated with skin temperature (*r* = 0.50). Other correlations should be interpreted with caution as these used heart rate in the calculation methods.

Between the pools of environmental and health variables, Pearson correlation coefficients of low to moderate strength were calculated (0.00 < *r* < 0.54) (Fig. 2, Supplementary Table 2). Relative humidity was negatively associated with heart rate (r = −0.30), while positive correlations were found between skin temperature and air temperature (r = 0.37), between skin temperature and solar radiation (r = 0.34), and between tympanic temperature and relative humidity (r = 0.30). Heat stress indices showed similar positive associations with skin temperature (0.43 < *r* < 0.54) and tympanic temperature (0.26 < *r* < 0.33) supporting the construct validity of the heat stress indices (Supplementary Table 2). Construct validity refers to how well an index measures an intended concept^24^. Nevertheless, the construct validity of heat stress indices was only partial, given the negative associations of heat stress indices with heart rate (− 0.22 < *r* < − 0.35), core temperature estimate (− 0.14 < *r* < − 0.30), and the Physiological Strain Index (− 0.18 < *r* − 0.32). However heart rate can also be affected by other factors such as psychological factors, physical activity, or work intensity and the latter was most likely influenced by behavioural thermoregulation at higher heat stress exposures. Overall, heat stress indices, as well as air temperature, black globe temperature, and solar radiation correlated most strongly with skin temperature.

### Independent and compound effects of heat stress on heat strain

The abovementioned relatively strong correlation of environmental variables with skin temperature was also apparent when assessing the independent effect of environmental variables on skin temperature through mixed effect linear models with random intercepts by study participant. First, we assessed the independent association of each environmental factor on the physiological variables. Here, increases in air temperature, relative humidity, and solar radiation led to highly robust increases in skin temperature, *ceteris paribus*, which was not found with air velocity, black globe temperature or metabolic rate (Table 2—Model 2A). Further, air temperature and relative humidity were associated with tympanic temperature (Table 2—Model 4A), and with estimated core temperature and the Physiological Strain Index as output variables, but the estimates were more imprecise (Table 2—Model 3A, 5A). However, we found no robust estimates for the association of environmental variables with heart rate (Table 2—Model 1A). On sensitivity testing, we found a robust estimate for increasing solar radiation in association with increased heart rate, whereby we rematched the highest 5-minute average heart rate within the 1-hour interval prior to each environmental datapoint (Supplementary Table 5). Sensitivity testing further showed that the models with water vapour pressure as an input variable (Supplementary Table 9—Models A) yielded comparable results as the models with relative humidity as an input variable (Table 2—Models A).

**Table 2:** Mixed effect linear models with random intercepts per participant.

	Model 1: heart rate	Model 2: skin temperature	Model 3: core temperature	Model 4: tympanic temperature	Model 5: physiological strain index
	Estimate (98% CI)	Estimate (98% CI)	Estimate (98% CI)	Estimate (98% CI)	Estimate (98% CI)
Model A—environmental parameters and physiological parameters					
Air temperature	0.59 (− 0.33; 1.50)	0.20 (0.14; 0.27)	1.91e^−2^ (− 4.92e^−3^; 4.28e^−2^)	0.05 (− 0.01; 0.11)	0.05 (− 0.03; 0.13)
Relative humidity	− 0.08 (− 0.20; 0.04)	0.02 (0.01; 0.03)	− 2.21e^−3^ (− 5.19e^−3^; 7.69e^−4^)	0.01 (2.36e^−3^; 0.01)	− 0.01 (− 0.02; 1.91e^−3^)
Air velocity	− 0.60 (− 2.26; 1.37)	− 0.05 (− 0.19; 0.09)	− 2.66e^−2^ (− 0.07; 2.20e^−2^)	0.01 (− 0.08; 0.11)	− 0.08 (− 0.24; 0.08)
Black globe temperature	0.20 (− 0.39; 0.78)	− 0.01 (− 0.05; 0.03)	2.38e^−3^ (− 0.01; 1.75e^−2^)	− 0.02 (− 0.07; 0.02)	0.01 (− 0.04; 0.06)
Solar radiation/100.000	− 0.02 (− 0.07; 0.02)	0.01 (2.78e^−3^; 0.01)	− 5.28e^−5^ (− 1.22e^−3^; 1.12e^−3^)	7.02e^−4^ (− 1.48e^−3^; 2.92e^−3^)	− 1.27e^−3^ (− 0.01; 2.59e^−3^)
Metabolic rate	− 0.03 (− 2.52; 2.45)	− 0.05 (− 0.22; 0.12)	1.38e^−2^ (− 0.06; 8.30e^−2^)	0.02 (− 0.09; 0.13)	0.06 (− 0.17; 0.28)
Model B—interaction between air temperature and relative humidity					
Air temperature	0.81 (0.35; 1.28)	0.20 (0.17; 0.23)	0.02 (0.01; 0.04)	0.02 (− 0.01; 0.05)	0.06 (0.02; 0.10)
Relative humidity	− 9.75 (− 60.49; 41.05)	− 2.59 (− 6.39; 1.24)	− 0.39 (− 1.62; 0.85)	3.46 (− 0.96; 7.91)	− 1.64 (− 5.71; 2.45)
Air temperature · relative humidity	0.29 (− 1.32; 1.89)	0.11 (0.01; 0.23)	0.01 (− 0.03; 0.05)	− 0.10 (− 0.24; 0.04)	0.05 (− 0.08; 0.18)
Model C—interaction between air temperature and gestational age					
Air temperature	0.55 (− 0.09; 1.20)	0.19 (0.14; 0.24)	0.02 (4.80e^−4^; 0.03)	− 0.03 (− 0.07; 0.01)	0.04 (− 0.02; 0.10)
Gestational age	− 16.36 (− 45.38; 13.05)	− 0.69 (− 2.97; 1.60)	− 0.52 (− 1.29; 0.26)	− 2.22 (− 4.22; − 0.30)	− 1.84 (− 4.39; 0.73)
Air temperature · gestational age	0.53 (− 0.34; 1.39)	0.01 (− 0.06; 0.07)	0.02 (− 6.88e^−3^; 0.04)	0.06 (3.12e^−3^; 0.12)	0.06 (− 0.02; 0.13)

Model A assessed the independent effect of each environmental variable on physiological variables (i.e. single models with environmental variables against each physiological variable). Model B assessed the interactive effect of air temperature and relative humidity on physiological variables, with air temperature as a continuous variable and relative humidity as a dummy variable at a 50% relative humidity threshold. Model C assessed the interactive effect of air temperature and gestational age on physiological variables, with air temperature as a continuous variable and gestational age as a dummy variable at a threshold of 27 gestational weeks, separating the second from the third trimester of pregnancy. *P*-values are in the Supplementary Table 4.

**Fig. 3:**
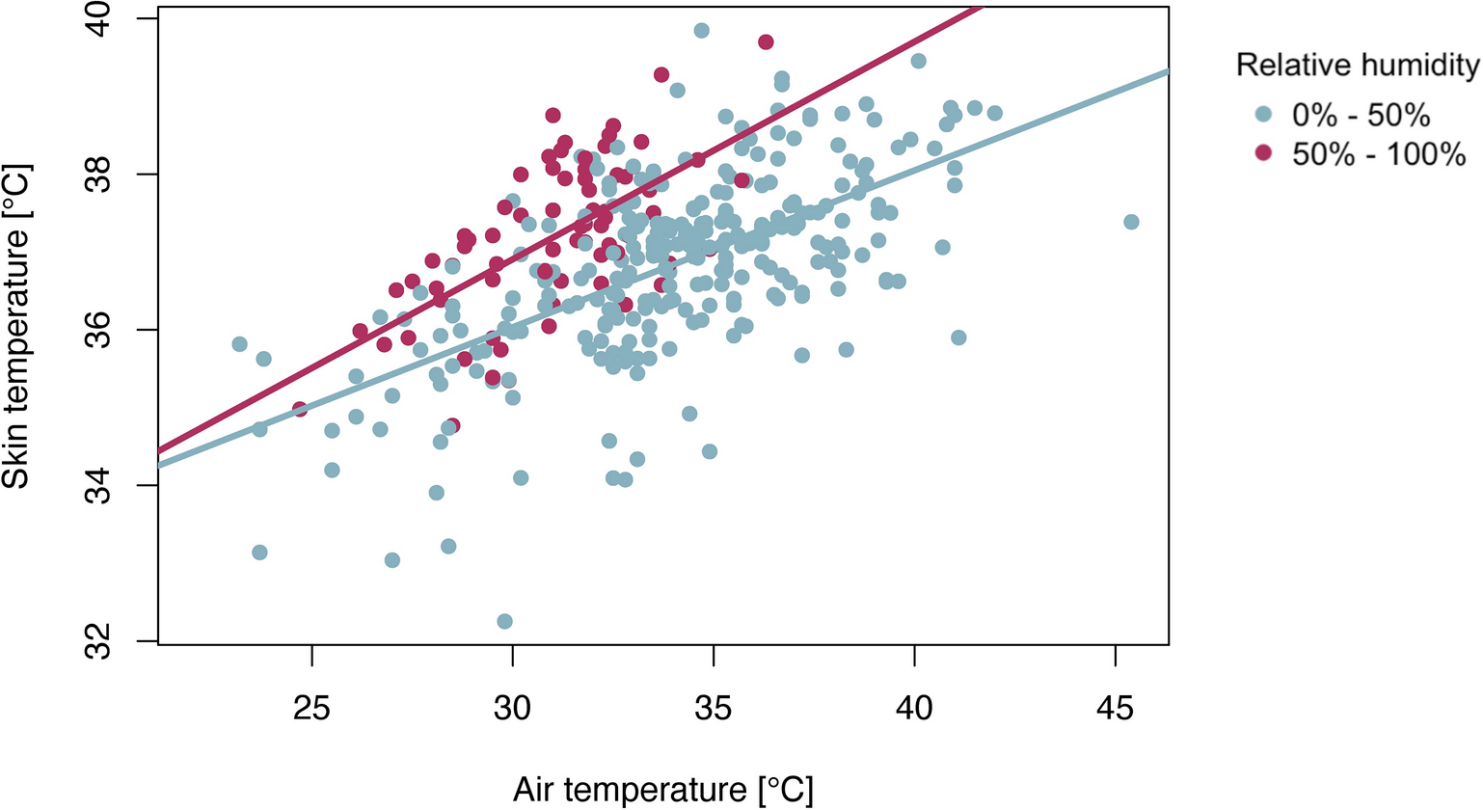
Interaction between air temperature and relative humidity in association with skin temperature at a threshold of 50% relative humidity. The skin temperature increases more rapidly with increasing temperature under conditions in which the relative humidity is above the 50% threshold.

Further interactive effects were found between air temperature and relative humidity in association with skin temperature (Table 2—Model 2B). Increases in skin temperature were more rapid when the air temperature rose under conditions of relative humidity above the 50% threshold (Fig. 3). On sensitivity testing, we did not detect an analogous interactive effect between air temperature and water vapour pressure in the association with skin temperature (Supplementary Table 9—Model 1B). Furthermore, no robust evidence for the interaction between air temperature and relative humidity was found in the association with heart rate, core temperature estimates, or tympanic temperature (Table 2—Model 1B, 3B, 4B, 5B).

Sensitivity analysis showed no confounding effect of fitness status or gestational age (Supplementary Tables 6 and 7). However, we found an interaction effect of gestational age with air temperature on all models (Table 2—Model 1C, 2C, 3C, 4C). With increasing temperature, women in their third trimester of pregnancy experienced greater increases in heart rate, skin temperature, estimated core temperature, and tympanic temperature than women in the second trimester of pregnancy did. This interaction effect of gestational age was also evident in the model with the Physiological Strain Index as the output variable (Table 2—Model 5D).

**Fig. 4:**
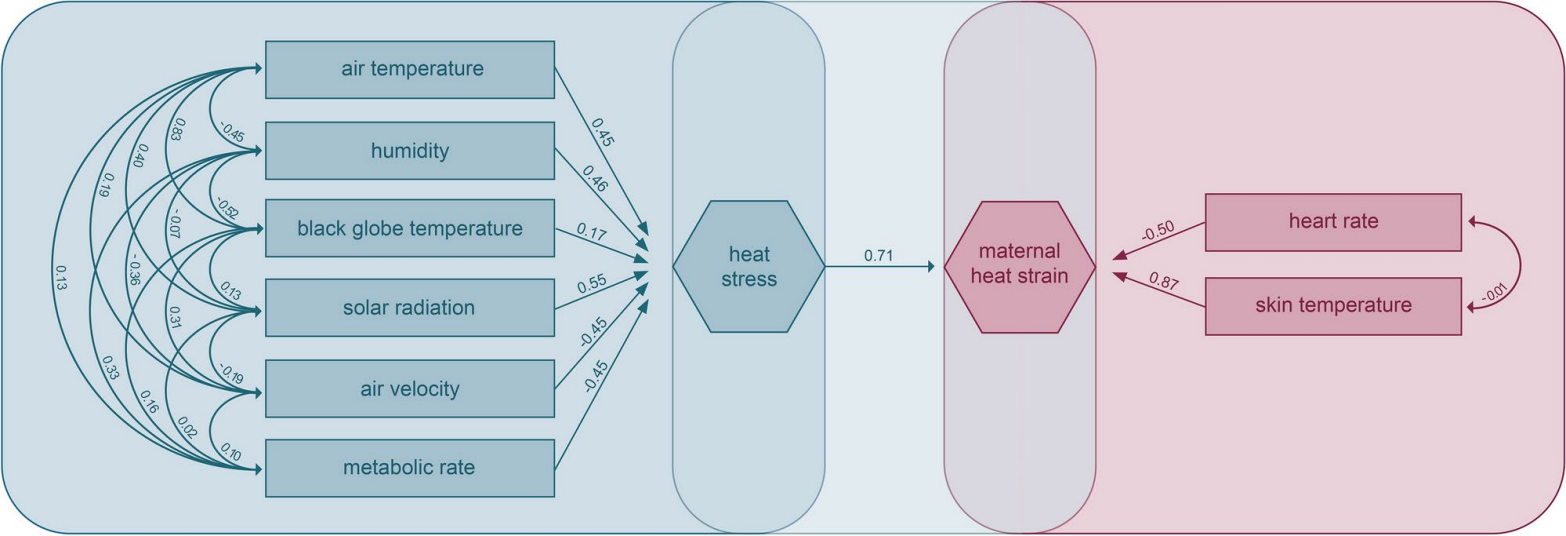
Composite confirmatory analysis with respective loadings of path coefficients between composite artefacts (heat stress and heat strain) and observable indicators (air temperature, relative humidity, black globe temperature, solar radiation, air velocity, metabolic rate, heart rate, skin temperature).

Through composite confirmatory analysis^25^, we assessed the simultaneous influence of environmental parameters on the conjunction of observed physiological parameters. We found a loading of 0.71 between heat stress and maternal heat strain (Fig. 4). On the one hand, heat stress was most strongly influenced by solar radiation (0.55), air temperature (0.45), and relative humidity (0.46). Factor loadings were relatively low for black globe temperature (0.17) and negative for air velocity (− 0.45) and metabolic rate (− 0.45). The factor loading of metabolic rate was negative because agricultural workers tend to reduce their level of activity when working in the heat (behavioural thermoregulation). On the other hand, heat strain was most strongly reflected in skin temperature with a factor loading of 0.87. The factor loading for heart rate was negative (− 0.50), most likely because the behavioural adaptations taken by study participants while working in the heat was not sufficiently captured by the estimated metabolic rate. In other words, pregnant women might have reduced their activity levels more than the estimated metabolic rate suggested and thus their heart rate decreased correspondingly. Even though the fit indices of our model lie within the optimal ranges (Table 3), the only robust estimates were for the loadings of air temperature, solar radiation, and skin temperature (Supplementary Table 7).

**Table 3:** Indices showing the model fit of the confirmatory composite analysis.

Assessment of model fit		
Indices	Optimal fitness	Obtained value
Chi square (X2/df)	1–3	1.28
Comparative fit index (CFI)	> 0.9	0.99
Root mean squared error of approximation (RMSEA)	< 0.1	0.06
Goodness-of-fit index (GFI)	> 0.9	0.95
Normed fit index (NFI)	> 0.9	0.97
Incremental fit index (IFI)	0–1	0.99

The table structure is based on the study of Yazdanirad et al.^26^.

## Discussion

Taken together, our findings demonstrate that physiological indicators of heat strain in pregnant women are significantly influenced by environmental conditions whilst working in the heat, particularly in the third trimester of pregnancy compared to the second. This study provides information on the complexity of analysing environmental parameters singly and could be used as evidence to support the adoption of heat stress indices for evaluating the pathophysiological effects of heat stress on pregnant women working in the heat.

We found that heat strain of pregnant women was more severe under conditions above a relative humidity of 50%, where the impact of rising air temperature on skin temperature increased at a greater rate in humid conditions. This result can add to the growing body of evidence related to the human adaptability limit to heat stress^18,27^, since robust field-based data on this is lacking and there remains uncertainty around stated thresholds^28,29^.

Furthermore, we found robust evidence for the increase in susceptibility to heat strain throughout pregnancy at rising temperatures across all physiological indicators, namely skin temperature, heart rate, tympanic temperature and estimated core temperature. More precisely, women in their third trimester of pregnancy experienced higher heat strain than women in their second trimester of pregnancy did. Given that the data in our study only covered the second and third trimester of pregnancy, it would be interesting for future research to compare these findings with data from early pregnancy. While our study covered the associations between exposure to extreme heat and maternal heat strain, there are similar results in the existing literature on the associations between exposure to extreme heat and adverse pregnancy outcomes. A recent study found that the third trimester of pregnancy is the most susceptible time for heat-related stillbirth^30^. A global analysis and meta-analysis detected an increased risk of preterm birth with an exposure to extreme heat during the last seven days of gestation^16,31^. However, another study has shown that the relative risk of heat-related stillbirth also increases in early pregnancy^32^.

Our study demonstrated the convergent validity of heat stress indices through correlation and construct validity with skin temperature and tympanic temperature. The Heat Index, UTCI, Apparent Temperature and WBGT were strongly associated with each other, despite incorporating different environmental input variables. Reassuringly, the selected heat stress indices showed similar associations with both skin temperature and tympanic temperature, which supports their applicability to our study setting. Therefore we recommend using these indices, such as the UTCI or WBGT in heat-health warning systems, given that these indices incorporate air temperature, relative humidity, solar radiation, and air velocity which showed associations to physiological variables in our study (Section “Independent and compound effects of heat stress on heat strain”). Although heat-health warning systems may be used as a public health measure to protect against heat-related health issues, there may be limitations to their practical use. We have previously found that complexities due to work demands and other socioeconomic pressures may limit behavioural options in our setting^33^. Alternative considerations could include activation of insurance schemes or unconditional cash transfers to protect women who work during pregnancy^34^.

There are six key limitations to our study. First, we tested the applicability of four out of more than 100 existing heat stress indices^35^. However, the Heat Index, Apparent Temperature, WBGT, and UTCI are indices that are often referred to in the literature^5^ and require input variables covered with our datasets, together with additional solar radiation modelling. We also tried to overcome this limitation by assessing the separate effects of environmental variables on maternal physiology, which increases the generalizability of the results. Second, given data availability constraints, our study design omitted variables such as clothing thermal insulation, systolic blood pressure, and diastolic blood pressure, which have been included in other studies^19,26^. Even though clothing thermal insulation represents an important factor in occupational heat exposure research, it was not measured in our study given that clothing characteristics were similar across study participants, and no participant was wearing microclimate cooling equipment or protective clothing. Furthermore, water intake, cloud cover, and socioeconomic factors could have been added to the models as confounders as they might have influenced our findings. Potentially, solar radiation could have influenced the measurement of skin temperature, given that the measurement devices were positioned on the upper portion of the back and although under clothing, would have been exposed to the sun while study participants bent at the waist to performed their agricultural tasks. WBGT measurements might not be fully accurate given that the HT200: heat stress WBGT meter is not a standard measurement device. Third, we acknowledge the potential imprecision of results sourced from the matching of datasets, given that the temporal resolution of the environmental dataset was in hourly intervals while the health dataset had a higher temporal resolution. For future research, it would be beneficial to have both environmental and health data points measured at the same time to maximize accuracy when determining the associations. We reached no clear conclusions about the effects of heat stress on heart rate and could not determine construct validity of heat stress indices on heart rate, core temperature estimates or the physiological strain index, possibly due to behavioural thermoregulation not captured in estimated metabolic rate. Even while applying a moving average merging technique (Supplementary Table 5) and confirmatory composite analysis (Supplementary Table 10), we found no robust estimates for the compound effects of air temperature and relative humidity on heart rate, possibly also due to unmeasured behavioural responses. Behavioural responses to extreme heat can include the shortening of work shifts or the reduction of work intensity. Given that the metabolic rate was estimated in our study and not directly measured with a device, these behavioural responses may not have been fully captured in our data analysis. This limitation might be resolved in future studies while measuring metabolic rate more effectively through a monitoring device to disentangle the effects of activity and heat stress. Fourth, our confirmatory composite analysis did not consider the effect of individual-level differences which may have arisen from measuring the physiological indicators of each study participant multiple times in each work shift. We considered each physiological datapoint independent of all other physiological datapoints. For future research, it would be beneficial grouping the datapoints per study participants in the confirmatory composite analysis. This would allow separation of the effect of environmental changes from the effect of individual-level differences. Such individual-level differences were only included in our mixed effect models with random intercepts. Fifth, we considered a linear association between variables and did not account for non-linearity since previous assessments with this data have shown that linear models had the best fit^1^. However, future analyses could explore non-linear associations in the relationship between humidity and air temperature to gain a more precise understanding of the interaction between variables^1^. Sixth, this study is based on a population of 92 pregnant farmers in West Kiang, The Gambia. Thus, results cannot directly be applied to wider study areas or study populations, especially in other climatic conditions.

Despite these limitations, our findings on the associations between environmental variables and physiological indicators of pregnant women in The Gambia can contribute to the development of climate change adaptation policies and offer pathways for further research in this field. Based on our results, implementing adaptation measures that reduce the exposure to heat stress on pregnant women working in agriculture would reduce physiological strain. The exposure to heat stress could be reduced with the establishment of heat-health warning systems, if these are linked to education and information campaigns on potential health threats. Ideally, heat-health warning systems would incorporate heat stress indices such as the UTCI and WBGT which have been tested within the local setting by the present study. Based on an hourly safety rating, agricultural workers could potentially adapt their schedules and work duration to minimize health hazards^7^. Additionally, the physiological impacts of heat stress on agricultural workers can be limited through protective clothing or microclimate cooling, which have been shown by previous research to significantly reduce the impacts of heat stress on the human body^36^, and could be tested in a pregnant population. A randomised controlled trial of separate and combined locally appropriate interventions would allow assessment of their effectiveness. Furthermore, it would be beneficial for further studies to link physiological heat strain to maternal health outcomes, including gestational diabetes or pre-eclampsia and to birth outcomes, such as stillbirth or preterm birth. Overall, policies that aim to protect maternal and newborn health in Africa should simultaneously address the individual, infrastructural, community and environmental factors to yield highest efficiency^3^. For optimal policy design and implementation, adaptive measures should be developed in close collaboration with the local community, and scientific investigations of the respective measures should be undertaken through a participatory research approach^37^.

## Methods

This study is based on datasets from a previous study by Bonell et al.^1^ and has applied the Pearson product moment correlation method, mixed effect models with random intercepts, and confirmatory composite analysis. The study population consisted of 92 pregnant women from West Kiang, The Gambia, who had been recruited through the local antenatal clinic and provided informed consent. Participants worked either on a small-scale farm, in agriculture or in a garden for more than 3 hours per day. Acutely ill participants, including those diagnosed with pre-eclampsia, eclampsia, gestational diabetes, or who had a history of heart disease were excluded from the study. A more detailed overview of the demographics, physical characteristics, and birth outcomes of the study participants is available in Supplementary Table 1. The study setting covers 9 villages within the West Kiang region: Jali, Janneh Kunda, Jiffarong, Kantong Kunda, Karantaba, Keneba, Kuli Kunda, Mandina, Manduar and Tankular. West Kiang is a district located in the Lower River Division of The Gambia and populated by 14,846 inhabitants. The main mode of subsistence is manual farming. Women work on average between 4.5 and 7.5 hours per day during pregnancy^38^.

Observational data (i) were collected in the study of Bonell et al.^1^ which measured both environmental and physiological indicators while study participants performed agricultural tasks outdoors within their work shift. There was a time gap of two months between work shifts of participants who were visited multiple times during pregnancy. Black globe temperature, dew point temperature, relative humidity, air temperature, and WBGT were collected using an HT200: heat stress WBGT meter. Air velocity was recorded with a portable Extech AN100 Thermo-Anemometer at 2 meters height. Skin temperature and maternal heart rate were measured with an Equivital LifeMonitor, which was placed at the back of the chest under the clothing. Core temperature was estimated based on skin temperature and heart rate. Metabolic rates were estimated through the matching of observed activity with indirect calorimetry data for energy expenditure^39^. The resolution of the datasets varied: Environmental parameters were available at hourly intervals, while physiological parameters were available at 5-minute intervals. Based on the environmental data points, we calculated heat stress indices with the R packages weathermetrics^40^, rBiometeo^41^ and HeatStress^42^. We calculated water vapour pressure (e [hPa]) based on relative humidity (rh [%]), saturated water vapour pressure (e_s_ [hPa]) and air temperature (T [°C]) with the following formula^43^:

$${\text{e }} = \frac{es \cdot rh}{{100}}{\text{with e}}_{{\text{s}}} = 6.11 \cdot 10 ^{{\frac{7.5 \cdot T}{{237.3 + T }}}}$$

The study of Bonell et al.^1^, on which this research project is based, has been approved by The Gambian Government and the Medical Research Council Unit The Gambia Joint Ethics Committee and the London School of Hygiene & Tropical Medicine Ethics Advisory Board in accordance with the Declaration of Helsinki^44^.

The retrieved modelled data (ii) from the ERA5 hourly land reanalysis dataset contains hourly surface net solar radiation for the geocodes of Jali, Janneh Kunda, Jiffarong, Kantong Kunda, Karantaba, Keneba, Kuli Kunda, Mandina, Manduar and Tankular^22^. In total, we extracted 80 subsets, each of which covered the modelled solar radiation for one village and month within the study period from August 2019 until March 2020. The datasets are temporally resolved at 1-hour intervals and spatially resolved on 0.09° horizontal and vertical grids. The surface net solar radiation is indicated in units of joules per square meter (J/m^2^) and represents the difference between the solar radiation reaching the Earth’s surface and the solar radiation reflected from the surface through the albedo effect^22^.

Given the varying time formats and resolutions of the abovementioned datasets (i-ii), we created uniform time stamps in POSIX.ct format and minimized the time differences between the data points to construct the final merged dataset. Every data point from the environmental dataset was matched with the average of each physiological variable over the previous hourly interval. We tested if short-term heart rate fluctuations which do not stem from heat stress have influenced our results while we rematched heart rate values with environmental data points through a 5-minute moving average interval. Observations were matched with the respective study participants’ gestational age, their result of a 6-minute walking test as an indication of fitness status, and the geographical location. We added respective estimated metabolic rates for both halves of the work shift. Each datapoint was matched with the modelled solar radiation from the ERA5 land datasets of the respective location and closest in time. The final merged dataset consisted of observational (i) and modelled data (ii). The data distribution was verified through descriptive statistics, more specifically, through summary statistics tables, boxplots, probability density functions, QQ plots, and Kolmogorov-Smirnov tests, serving as a basis for outlier detection. We removed outliers of heart rate below 60 bpm, above 200 bpm, or below a confidence interval of 85%, as well as of skin temperature, which was three standard deviations away from the mean. The environmental data points remained complete.

Through the Pearson product moment correlation method we assessed the separate associations between variables as a linear relationship of a strength between − 1 and 1. We determined the Pearson product moment correlation coefficient r for each variable in relation with all other variables^45^. This methodology also served to determine the convergent and construct validity of heat stress indices. Construct validity was tested while computing the correlation coefficient r between each heat stress index and the physiological variables. Convergent validity was tested while computing the correlation coefficient r between heat stress indices. Previous studies that have validated heat stress indices in the context of male farmers and mine workers in Iran have applied a similar methodology^1,19^. We assessed not only associations of environmental parameters with separate health variables, but also with a modified version of the physiological strain index, which is a composite index based on skin temperature, tympanic temperature, and heart rate. The advantage of the modified version of the physiological strain index is that it does not require core temperature measurements. Core temperature measurements were not available in our dataset due to safety constraints associated with pregnancy^1,46^.

Through mixed effect models with random intercepts, we assessed the independent and linear effects of the environmental variables on the physiological variables. We computed three types of models, namely (A) a model for each physiological variable as an output variable with the environmental variables as input variables, (B) a model for each physiological variable as an output variable with air temperature, humidity, and an interactive term between air temperature and humidity as input variables to test for compound effects, and (C) a model for each physiological variable as an output variable with air temperature, gestational age and an interactive term between air temperature and gestational age as input variables to test for compound effects. We added a random intercept for each study participant to the models. This methodology allowed us to account for individual differences between study participants. To detect multicollinearity we computed the variance inflation factor^47^. We applied sensitivity analysis to assess potential confounding of gestational age and fitness status in the association between environmental variables and physiological variables. This was done by including gestational age and fitness status as additional linear model parameters into models and verifying if estimates of all other parameters changed in comparison to the standard models without gestational age and fitness status. Furthermore, we applied sensitivity analysis to assess how our model changed when we used water vapour pressure instead of relative humidity as an input variable into the mixed effect models with random intercepts.

Through the confirmatory composite analysis, we modelled the simultaneous interplay between multiple environmental and multiple physiological variables^25^. For this purpose we used all complete cases of our dataset and removed all rows with missing variables. We constructed a model with air temperature, relative humidity, black globe temperature, solar radiation, air velocity, and metabolic rate as observable indicators of the composite artefact of heat stress, and with heart rate and skin temperature as observable indicators of the composite artefact of maternal heat strain. We used the bootstrap resampling method and computed the factor loading for each relationship of the environmental variables with the composite artefact of heat stress, between each physiological variable and the composite artefact of maternal heat strain, and between both composite artefacts. The results from the mixed effect models with random intercepts were compared to those from to enhance the robustness of our findings and assess the interplay between variables.

## Supplementary Information

Supplementary Information.

## Data Availability

Anonymized data is available from the LSHTM online data repository: https://datacompass.lshtm.ac.uk/id/eprint/3390/.
