# Supplementary material for: Integrating observational and modelled data to advance the understanding of heat stress effects on pregnant subsistence farmers in the gambia
Source: Sci Rep. 2024 Oct 23;14:24977. doi: 10.1038/s41598-024-74614-y (PMC11499601; doi:10.1038/s41598-024-74614-y)
Supplement: Supplementary file 1 — Supplementary Information. [file 41598_2024_74614_MOESM1_ESM.docx]

# Supplementary Figures

**Supplementary Figure 1.** Scatterplots showing the separate associations between health variables and environmental variables. Each environmental data point was merged with the previous hourly average of the physiological variables.

**Supplementary Figure 2.** Scatterplots showing the separate associations between environmental variables and heart rate, dependent on different merging techniques. Each environmental data point was merged with the highest value of all 5-minute averages of heart rate within the previous hourly intervals *(Supplementary Figure 1)*

Gestational age group

**Supplementary Figure 3.** Interaction effect between air temperature and gestational age on the association with health variables at a threshold of 27 gestational weeks, separating the second from third trimester of pregnancy.

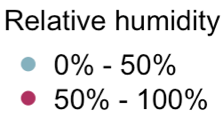


Water vapour pressure

0-21 hPa

21-50 hPa

**Supplementary Figure 4.** Association between air temperature and skin temperature, grouped by the level of water vapour pressure at a threshold of 21 hPa. No significant interactive effect between air temperature and water vapour pressure could be detected in association with skin temperature.

# Supplementary Tables

|  | **Participants (n = 92)** |
| --- | --- |
| Demographic parameters | |
| Age, years | 27.7 (23.7–35.8) |
| Schooling, years in education | 5 (4–8) |
| Ethnicity | |
| Mandinka | 85 (92%) |
| Wolof | 6 (7%) |
| Fula | 1 (1%) |
| Occupation | |
| Farmer | 74 (80%) |
| Other | 18 (20%) |
| Marital status | |
| Single | 2 (2%) |
| Married | 89 (97%) |
| Widowed | 1 (1%) |
| Obstetric history | |
| Gravida | 4 (2–7) |
| Parity | 3 (1–5.25) |
| Gestational age at visit, weeks | 28.5 (23.6 - 32.9) |
| Weight | |
| Median, kg | 61.9 (55.8–97.3) |
| BMI, kg/m2 | 23.0 (21.3–25.9) |
| Mid-upper arm circumference, cm | 27.0 (25.1–29.7) |
| Percentage fat mass, % | 28.7% (25.8–33.8) |
| Height, cm | 162.9 (5.6) |
| Birth outcome | |
| Normal birth | 53/91 (58%)* |
| Small for gestational age | 24/91 (26%)* |
| Preterm | 12/92 (13%) |
| Low birthweight | 12/91 (13%)* |
| Stillbirth or intrapartum death | 3/92 (3%) |

**Supplementary Table 1.** Demographics, physical characteristics,

and birth outcomes of participants. Reprinted from Bonell et al. ^1^

| **r-values** | Air temperature | Relative humidity | Black globe temperature | Air velocity | Solar radiation | Heat index | Universal Thermal Climate Index | Apparent Temperature | Wet Bulb Globe Temperature | Heart rate | Skin temperature | Core temperature estimate | Tympanic temperature | Physiological Strain index |
| --- | --- | --- | --- | --- | --- | --- | --- | --- | --- | --- | --- | --- | --- | --- |
| Air temperature | 1.00 | -0.44 . | 0.80 ** | 0.13 | 0.42 . | 0.34 . | 0.81 ** | 0.42 . | 0.11 | 0.00 | 0.37 . | 0.06 | 0.07 | 0.03 |
| Relative humidity | -0.44 . | 1.00 | -0.46 . | -0.35 . | -0.08 | 0.65 * | 0.16 | 0.60 * | 0.82 ** | -0.30 . | 0.22 | -0.29 | 0.30 . | -0.30 . |
| Black globe temperature | 0.80 ** | -0.46 . | 1.00 | 0.25 | 0.11 | 0.24 | 0.59 * | 0.23 | 0.03 | 0.00 | 0.18 | 0.03 | -0.09 | 0.02 |
| Air velocity | 0.13 | -0.35 . | 0.25 | 1.00 | -0.22 | -0.23 | -0.11 | -0.39 . | -0.30 . | 0.25 | -0.25 | 0.25 | -0.13 | 0.25 |
| Solar radiation | 0.42 . | -0.08 | 0.11 | -0.22 | 1.00 | 0.19 | 0.39 . | 0.33 . | 0.16 | -0.16 | 0.34 . | -0.09 | 0.18 | -0.12 |
| Heat index | 0.34 . | 0.65 * | 0.24 | -0.23 | 0.19 | 1.00 | 0.80 ** | 0.94 *** | 0.91 *** | -0.35 . | 0.43 . | -0.29 | 0.30 . | -0.32 . |
| Universal Thermal Climate Index | 0.81 ** | 0.16 | 0.59 * | -0.11 | 0.39 . | 0.80 ** | 1.00 | 0.86 ** | 0.65 ** | -0.22 | 0.54 * | -0.14 | 0.26 | -0.18 |
| Apparent Temperature | 0.42 . | 0.60 * | 0.23 | -0.39 . | 0.33 . | 0.94 *** | 0.86 ** | 1.00 | 0.92 *** | -0.33 . | 0.54 * | -0.27 | 0.33 . | -0.30 . |
| Wet Bulb Globe Temperature | 0.11 | 0.82 ** | 0.03 | -0.30 . | 0.16 | 0.91 *** | 0.65 ** | 0.92 *** | 1.00 | -0.34 . | 0.44 . | -0.30 . | 0.31 . | -0.32 . |
| Heart rate | 0.00 | -0.30 | 0.00 | 0.25 | -0.16 | -0.35 . | -0.22 | -0.33 . | -0.34 . | 1.00 | 0.02 | 0.97 *** | -0.05 | 0.99 *** |
| Skin temperature | 0.37 . | 0.22 | 0.18 | -0.25 | 0.34 . | 0.43 . | 0.54 * | 0.54 * | 0.44 . | 0.02 | 1.00 | 0.10 | 0.50 * | 0.06 |
| Core temperature estimate | 0.06 | -0.29 | 0.03 | 0.25 | -0.09 | -0.29 | -0.14 | -0.27 | -0.30 . | 0.97 *** | 0.10 | 1.00 | -0.01 | 0.99 *** |
| Tympanic temperature | 0.07 | 0.30 . | -0.09 | -0.13 | 0.18 | 0.30 . | 0.26 | 0.33 . | 0.31 . | -0.05 | 0.50 * | -0.01 | 1.00 | -0.03 |
| Physiological Strain index | 0.03 | -0.30 . | 0.02 | 0.25 | -0.12 | -0.32 . | -0.18 | -0.30 . | -0.32 . | 0.99 *** | 0.06 | 0.99 *** | -0.03 | 1.00 |

**Supplementary Table 2.** Pearson correlation matrix of environmental and health parameters. The items were denoted as « » for neglectable correlation coefficients (±0.00 to ±0.30), « . » for low correlations (±0.30 to ±0.50), « * » for moderate correlations (±0.50 to ±0.70), « ** » for high correlations (±0.70 to ±0.90), and « *** » for very high correlations (±0.90 to ±1.00) ^23^. The matrix was separated by the pool of environmental variables in the upper part and the pool of physiological variables in the lower part.

| **p-values** | Air temperature | Relative humidity | Black globe temperature | Air velocity | Solar radiation | Heat index | Universal Thermal Climate Index | Apparent Temperature | Wet Bulb Globe Temperature | Heart rate | Skin temperature | Core temperature estimate | Tympanic temperature | Physiological Strain index |
| --- | --- | --- | --- | --- | --- | --- | --- | --- | --- | --- | --- | --- | --- | --- |
| Air temperature |  | 0.00 | 0.00 | 0.22 | 0.00 | 0.99 | 0.00 | 0.55 | 0.51 | 0.75 | 0.00 | 0.00 | 0.00 | 0.31 |
| Relative humidity | 0.00 |  | 0.00 | 0.00 | 0.45 | 0.00 | 0.04 | 0.01 | 0.00 | 0.00 | 0.00 | 0.14 | 0.00 | 0.00 |
| Black globe temperature | 0.00 | 0.00 |  | 0.02 | 0.29 | 0.97 | 0.10 | 0.77 | 0.41 | 0.88 | 0.03 | 0.00 | 0.03 | 0.76 |
| Air velocity | 0.22 | 0.00 | 0.02 |  | 0.04 | 0.02 | 0.02 | 0.02 | 0.24 | 0.02 | 0.03 | 0.33 | 0.00 | 0.00 |
| Solar radiation | 0.00 | 0.45 | 0.29 | 0.04 |  | 0.14 | 0.00 | 0.40 | 0.09 | 0.25 | 0.07 | 0.00 | 0.00 | 0.13 |
| Heat index | 0.99 | 0.00 | 0.97 | 0.02 | 0.14 |  | 0.87 | 0.00 | 0.63 | 0.00 | 0.00 | 0.04 | 0.00 | 0.00 |
| Universal Thermal Climate Index | 0.00 | 0.04 | 0.10 | 0.02 | 0.00 | 0.87 |  | 0.35 | 0.00 | 0.57 | 0.00 | 0.00 | 0.00 | 0.00 |
| Apparent Temperature | 0.55 | 0.01 | 0.77 | 0.02 | 0.40 | 0.00 | 0.35 |  | 0.89 | 0.00 | 0.01 | 0.19 | 0.01 | 0.00 |
| Wet Bulb Globe Temperature | 0.51 | 0.00 | 0.41 | 0.24 | 0.09 | 0.63 | 0.00 | 0.89 |  | 0.76 | 0.00 | 0.01 | 0.00 | 0.00 |
| Heart rate | 0.75 | 0.00 | 0.88 | 0.02 | 0.25 | 0.00 | 0.57 | 0.00 | 0.76 |  | 0.00 | 0.09 | 0.00 | 0.00 |
| Skin temperature | 0.00 | 0.00 | 0.03 | 0.03 | 0.07 | 0.00 | 0.00 | 0.01 | 0.00 | 0.00 |  | 0.00 | 0.00 | 0.00 |
| Core temperature estimate | 0.00 | 0.14 | 0.00 | 0.33 | 0.00 | 0.04 | 0.00 | 0.19 | 0.01 | 0.09 | 0.00 |  | 0.00 | 0.00 |
| Tympanic temperature | 0.00 | 0.00 | 0.03 | 0.00 | 0.00 | 0.00 | 0.00 | 0.01 | 0.00 | 0.00 | 0.00 | 0.00 |  | 0.00 |
| Physiological Strain index | 0.31 | 0.00 | 0.76 | 0.00 | 0.13 | 0.00 | 0.00 | 0.00 | 0.00 | 0.00 | 0.00 | 0.00 | 0.00 |  |

**Supplementary Table 3.** P-values of Pearson correlation matrix of environmental and health parameters.

|  | **Model 1:**  **Heart rate** | **Model 2: Skin**  **temperature** | **Model 3: Core**  **temperature** | **Model 4: Tympanic temperature** | **Model 5: Physiological**  **Strain Index** |
| --- | --- | --- | --- | --- | --- |
|  | p-value | p-value | p-value | p-value | p-value |
| *Model A – environmental parameters and physiological parameters* | | | | | |
| Air temperature | 0.14 | 1.25e-11 | 0.06 | 0.05 | 0.14 |
| Relative humidity | 0.11 | 1.91e-7 | 0.09 | 1.15e-3 | 0.06 |
| Air velocity | 0.47 | 0.40 | 0.21 | 0.78 | 0.25 |
| Black globe temperature | 0.44 | 0.51 | 0.72 | 0.20 | 0.64 |
| Solar radiation / 100.000 | 0.24 | 2.56e-5 | 0.92 | 0.47 | 0.44 |
| Metabolic rate | 0.98 | 0.50 | 0.65 | 0.67 | 0.57 |
| *Model B – interaction between air temperature and relative humidity* | | | | | |
| Air temperature | 5.7e-5 | 2.00e-16 | 2.97e-5 | 0.12 | 1.21e-3 |
| Relative humidity | 0.66 | 0.116 | 0.47 | 0.08 | 0.35 |
| Air temperature · relative humidity | 0.68 | 0.033 | 0.50 | 0.11 | 0.39 |
| *Model C – interaction between air temperature and gestational age* | | | | | |
| Air temperature | 0.05 | 2.87e^-16^ | 0.02 | 0.13 | 0.10 |
| Gestational age | 0.19 | 0.48 | 0.12 | 0.01 | 0.09 |
| Air temperature · gestational age | 0.14 | 0.81 | 0.10 | 0.02 | 0.07 |

**Supplementary Table 4.** P-values of mixed effect linear models with random intercepts per participant. Model outputs are in Table 2.

|  | **(a) Model 1:**  **Heart rate** | |  | **(b) Model 1:**  **Re-matched heart rate** | |
| --- | --- | --- | --- | --- | --- |
|  | Estimate  (95% CI) | p-value |  | Estimate  (95% CI) | p-value |
| *Model A – environmental parameters and physiological parameters* | | | | | |
| Air temperature | 0.59  (-0.19 ; 1.35) | 0.14 |  | 0.37  (-0.44 ; 1.18) | 0.37 |
| Relative humidity | -0.08  (-0.18 ; 0.02) | 0.11 |  | -0.08  (-0.19 ; 0.04) | 0.19 |
| Air velocity | -0.60  (-2.25 ; 1.05) | 0.47 |  | -0.12  (-1.81 ; 1.58) | 0.89 |
| Black globe temperature | 0.20  (-0.29 ; 0.68) | 0.44 |  | 0.21  (-0.30 ; 0.72) | 0.42 |
| Solar radiation e^5^ | -0.02  (-0.06 ; 0.02) | 0.24 |  | -0.05  (-0.09 ; -4.21e^-3^) | 0.03 |
| Metabolic rate | -0.03  (-2.12 ; 2.06) | 0.98 |  | 0.01  (-2.33 ; 2.35) | 0.99 |
| AIC | 2414.48 | - |  | 2360.86 | - |

**Supplementary Table 5.** Mixed effect models with random intercepts comparing two different merging techniques. (a) Each environmental data point was merged with the previous hourly average of heart rate. (b) Each environmental data point was merged with the highest value of all 5-minute averages of heart rate within the previous hourly interval.

|  | **Model 1:**  **Heart rate** | | **Model 2: Skin**  **temperature** | | **Model 3: Core**  **temperature** | | **Model 4: Tympanic temperature** | | **Model 5: Physiological**  **Strain Index** | |
| --- | --- | --- | --- | --- | --- | --- | --- | --- | --- | --- |
|  | Estimate  (95% CI) | p-value | Estimate  (95% CI) | p-value | Estimate  (95% CI) | p-value | Estimate  (95% CI) | p-value | Estimate  (95% CI) | p-value |
| *Model A – environmental parameters and physiological parameters* | | | | | | | | | | |
| Air temperature | 0.54  (-0.24 ; 1.31) | 0.17 | 0.20  (0.15 ; 0.26) | 6.86e-12 | 0.02  (-7.69e-4 ; 0.04) | 0.06 | 0.05  (2.73e^-3^ ; 0.10) | 0.05 | 0.05  (-0.01 ; 0.11) | 0.12 |
| Relative humidity | -0.06  (-0.16 ; 0.04) | 0.24 | 0.02  (0.01 ; 0.03) | 1.03e-6 | -1.50e^-3^  (-3.99e^-3^ ; 1.01e^-3^) | 0.25 | 0.01  (2.80e^-3^ ; 0.01) | 2.57e-3 | -5.50e^-3^  (-0.01 ; 2.75e^-3^) | 0.20 |
| Air velocity | -0.51  (-2.14 ; 1.16) | 0.54 | -0.06  (-0.19 ; 0.06) | 0.34 | -0.02  (-0.06 ; 0.02) | 0.23 | 0.01  (-0.07 ; 0.09) | 0.78 | -0.07  (-0.21 ; 0.06) | 0.28 |
| Black globe temperature | 0.19  (-0.29 ; 0.69) | 0.43 | -0.01  (-0.05 ; 0.02) | 0.50 | 2.20e-3  (1.03e^-3^ ; 0.01) | 0.74 | -0.02  (-0.06 ; 0.01) | 0.20 | 0.01  (-0.03 ; 0.05) | 0.66 |
| Solar radiation e^5^ | -0.02  (-0.05 ; 0.02) | 0.29 | 0.01  (3.23e^-3^ ; 8.69e^-3^) | 3.18e-5 | -6.12e^-5^  (-1.03e^-3^ ; 9.09e^-4^) | 0.90 | 7.25e-4  (-1.10e^-3^ ; 2.60e^-3^) | 0.45 | -1.29e^-3^  (-4.47 ; 1.91e^-3^) | 0.43 |
| Metabolic rate | 0.21  (-1.84 ; 2.28) | 0.84 | -0.05  (-0.20 ; 8.95e^-2^) | 0.48 | 2.15e^-2^  (-0.04 ; 0.08) | 0.47 | 0.02  (-0.07 ; 0.11) | 0.66 | 0.08  (-0.10 ; 0.26) | 0.40 |
| Fitness level | 0.03  (1.48e^-3^ ; 0.06) | 0.05 | -1.64e-3  (-3.78e^-3^ ; 4.85e^-4^) | 0.13 | 1.15e^-3^  (3.02e^-4^ ; 1.99e^-3^) | 0.01 | -2.72e^-4^  (-1.77e^-3^ ; 1.23e^-3^) | 0.73 | 3.88e^-3^  (1.10e^-3^ ; 0.01) | 0.01 |

**Supplementary Table 6.** Mixed effect models with random intercepts with fitness status as an additional variable were used to verify potential confounding effects.

|  | **Model 1:**  **Heart rate** | | **Model 2: Skin**  **temperature** | | **Model 3: Core**  **temperature** | | **Model 4: Tympanic temperature** | | **Model 5: Physiological**  **Strain Index** | |
| --- | --- | --- | --- | --- | --- | --- | --- | --- | --- | --- |
|  | Estimate  (95% CI) | p-value | Estimate  (95% CI) | p-value | Estimate  (95% CI) | p-value | Estimate  (95% CI) | p-value | Estimate  (95% CI) | p-value |
| *Model A – environmental parameters and physiological parameters* | | | | | | | | | | |
| Air temperature | 0.59  (-0.19 ; 1.36) | 0.14 | 0.20  (0.14 ; 0.25) | 2.18e-11 | 1.93e^-2^  (-1.08e^-3^ ; 3.93e^-2^) | 0.06 | 0.04  (-0.01 ; 0.09) | 0.10 | 0.05  (-0.02 ; 0.12) | 0.14 |
| Relative humidity | -0.08  (-0.19 ; 0.02) | 0.12 | 0.02  (0.01 ; 0.02) | 2.95e-6 | -2.17e-3  (-4.74e^-3^ ; 4.08e^-4^) | 0.11 | 0.01  (2.86e^-3^ ; 0.01) | 2.03e-3 | -7.83e^-3^  (-0.02 ; 0.12) | 0.08 |
| Air velocity | -0.61  (-2.25 ; 1.05) | 0.47 | -0.06  (-0.18 ; 0.06) | 0.33 | -2.65e^-2^  (-0.07 ; 1.44e^-2^) | 0.21 | 0.01  (-0.07 ; 0.09) | 0.78 | -0.08  (-0.21 ; 0.05) | 0.25 |
| Black globe temperature | 0.20  (-0.29 ; 0.69) | 0.43 | -0.01  (-0.04 ; 0.02) | 0.63 | 2.31e^-3^  (-0.01 ; 1.51) | 0.72 | -0.02  (-0.05 ; 0.02) | 0.31 | 9.77e^-3^  (-0.03 ; 0.05) | 0.65 |
| Solar radiation e^5^ | -0.02  (-0.06 ; 0.02) | 0.23 | 0.01  (3.11e-3 ; 0.01) | 4.42e-5 | -5.28e^-5^  (-1.03e^-2^ ; 1.51e^-2^) | 0.92 | 7.16e^-4^  (-1.07e^-3^ ; 2.56e^-3^) | 0.46 | -1.28e^-3^  (-4.51e^-3^ ; 1.99e^-3^) | 0.44 |
| Metabolic rate | -0.05  (-2.14 ; 2.06) | 0.96 | -0.06  (-0.20 ; 0.08) | 0.43 | 1.35e^-2^  (-0.04 ; 7.19e^-2^) | 0.65 | 0.02  (-0.07 ; 0.11) | 0.67 | 0.06  (-0.14 ; 0.25) | 0.58 |
| Gestational age | -0.02  (-0.28 ; 0.25) | 0.90 | -0.02  (-0.04 ; -0.01) | 0.01 | 5.03e^-4^  (-0.01 ; 7.45e^-3^) | 0.89 | -0.01  (-0.01 ; 1.69e^-3^) | 0.11 | 1.18e^-3^  (-0.02 ; 0.02) | 0.92 |

**Supplementary Table 7.** Mixed effect models with random intercepts and gestational age as an additional variable were used to verify potential confounding.

|  | **Model 1:**  **Heart rate** | **Model 2: Skin**  **temperature** | **Model 3: Core**  **temperature** | **Model 4: Tympanic temperature** | **Model 5: Physiological**  **Strain Index** |
| --- | --- | --- | --- | --- | --- |
|  | vif | vif | vif | vif | vif |
| *Model A – environmental parameters and physiological parameters* | | | | | |
| Air temperature | 4.19 | 4.23 | 3.81 | 4.48 | 3.81 |
| Relative humidity | 1.67 | 1.69 | 1.73 | 1.53 | 1.73 |
| Air velocity | 1.13 | 1.13 | 1.17 | 1.14 | 1.21 |
| Black globe temperature | 3.40 | 3.49 | 3.03 | 4.09 | 3.03 |
| Solar radiation e^5^ | 1.58 | 1.56 | 1.61 | 1.43 | 1.62 |
| Metabolic rate | 1.10 | 1.10 | 1.16 | 1.08 | 1.16 |

**Supplementary Table 8.** Variance inflation factors (vif) of mixed effect models with random intercepts. The variables are denoted as « » for neglectable variance inflation factors (0.00 < vif < 5.00), « * » for high variance inflation factors that might be problematic (5.00 < vif < 10.00), and « ** » for variance inflation factors showing high signs of multicollinearity (10.00 < vif).

|  | **Model 1:**  **Heart rate** | **Model 2: Skin**  **temperature** | **Model 3: Core**  **temperature** | **Model 4: Tympanic temperature** | **Model 5: Physiological**  **Strain Index** |
| --- | --- | --- | --- | --- | --- |
|  | Estimate  (98% CI) | Estimate  (98% CI) | Estimate  (98% CI) | Estimate  (98% CI) | Estimate  (98% CI) |
| *Model A – environmental parameters and physiological parameters* | | | | | |
| Air temperature | 0.68  (-0.24 ; 1.58) | 0.18  (0.12 ; 0.25) | 2.19e^-2^  (-1.97e^-3^ ; 0.05) | 0.05  (-0.01 ; 0.11) | 0.06  (-0.02 ; 0.14) |
| Water vapour pressure | -0.19  (-0.43 ; 0.06) | 0.04  (0.02 ; 0.06) | -5.02e^-3^  (-0.01 ; 1.16e^-3^) | 0.01  (2.96e^-3^ ; 0.02) | -0.02  (-0.04 ; 2.5e^-3^) |
| Air velocity | -0.66  (-2.62 ; 1.31) | -0.05  (-0.19 ; 0.10) | -2.81e^-2^  (-0.08 ; 0.02) | 0.01  (-0.09 ; 0.11) | -0.08  (-0.24 ; 0.07) |
| Black globe temperature | 0.18  (-0.40 ; 0.77) | -0.01  (-0.05 ; 0.03) | 1.92e^-3^  (-0.01 ; 0.02) | -0.03  (-0.07 ; 0.02) | 0.01  (-0.04 ; 0.06) |
| Solar radiation / 100.000 | -0.02  (-0.07 ; 0.02) | 0.01  (2.53e^-2^ ; 0.01) | -2.61e^-5^  (-1.19e^-3^ ; 1.14e^-3^) | 6.19e^-4^  (-1.59e^-3^ ; 2.89e^-3^) | -1.18e^-3^  (-0.01 ; 2.68e^-3^) |
| Metabolic rate | 1.11 e^-3^  (-2.46 ; 2.45) | -0.06  (-0.23 ; 0.10) | 1.40e^-2^  (-0.05 ; 0.08) | 0.01  (-0.10 ; 0.12) | 0.06  (-0.17 ; 0.28) |
| *Model B – interaction between air temperature and water vapour pressure* | | | | | |
| Air temperature | 0.71 (0.12 ; 1.29) | 0.19  (0.14 ; 0.23) | 0.02  (4.44e^-3^ ; 0.04) | 0.03  (-0.01 ; 0.07) | 0.05  (-4.02e^-4^ ; 0.11) |
| Water vapour pressure | -3.14  (-32.96 ; 26.64) | -1.05 (-3.41 ; 1.35) | -0.26  (-1.07 ; 0.55) | 2.28  (-0.13 ; 4.73) | -0.79  (-3.46 ; 1.89) |
| Air temperature · water vapour pressure | -0.01  (-0.90 ; 0.88) | 0.05 (-0.02 ; 0.11) | 0.01  (-0.02 ; 0.03) | -0.06  (-0.13 ; 0.01) | 0.02  (-0.06 ; 0.09) |

**Supplementary Table 9.** Mixed effect models with random intercepts with water vapour pressure instead of humidity.

| **Confirmatory composite analysis** | | | |  |
| --- | --- | --- | --- | --- |
| *Effect on heat stress* | | | |  |
|  |  | Loading  (95% CI) | p-value |  |
| Air temperature |  | 0.45  (-0.38 ; 0.68) | 0.08 |  |
| Relative humidity |  | 0.46  (-0.61 ; 0.67) | 0.15 |  |
| Air velocity |  | -0.45  (-0.68 ; 0.59) | 0.15 |  |
| Black globe temperature |  | 0.17  (-0.21 ; 0.42) | 0.29 |  |
| Solar radiation |  | 0.54  (-0.54 ; 0.73) | 0.09 |  |
| Metabolic rate |  | -0.45  (-0.65 ; 0.59) | 0.14 |  |
| *Effect on heat strain* | | | | |
| Heart rate |  | -0.50  (-0.71 ; 0.69) | 0.16 |  |
| Skin temperature |  | 0.87  (-0.84 ; 0.977) | 0.08 |  |
| Heat stress |  | 0.71  (-0.72 ; 0.83) | 0.07 |  |

**Supplementary Table 10.** Results from confirmatory composite analysis. Loadings between observed variables (air temperature, relative humidity, air velocity, black globe temperature, solar radiation, heart rate and skin temperature) and composite artefacts (heat stress and heat strain).
